# Clinical Profile and Outcome of Adult Patients with Scrub Typhus in a Tertiary Care Centre of Eastern Nepal

**DOI:** 10.31729/jnma.4777

**Published:** 2020-03-31

**Authors:** Ram Hari Ghimire, Ashima Ghimire, Sunil Dhungana, Bides Bista, Rupesh Kumar Shreewastav

**Affiliations:** 1 Department of Pulmonary, Critical Care and Sleep Medicine, Nobel Medical College Teaching Hospital, Biratnagar, Nepal; 2 Department of Biochemistry, Nobel Medical College Teaching Hospital, Biratnagar, Nepal

**Keywords:** *diagnosis*, *Nepal*, *scrub typhus*

## Abstract

**Introduction:**

Scrub typhus is an under-diagnosed and undertreated zoonotic human infection. There are no data related to profile of adult patients in Nepal. We conducted this study to report socio-demographic, clinical profile and complications of scrub typhus in our scenario.

**Methods:**

This was a descriptive cross-sectional study carried out in Nobel Medical College Teaching Hospital, eastern Nepal. The sample enrollment process was consecutively who were admitted under medical ward and intensive care unit. Diagnosis was established serologically with positive test of IgM antibodies against scrub typhus using immuno-chromatography. Operational definitions for organ system dysfunction were based upon simple available clinic laboratory profiles and imaging. Collected data were entered in Microsoft Excel 2007 and converted it into Statistical Package for Social Science 11.5 Version for statistical analysis.

**Results:**

A total of 47 patients were analyzed during this study. Diagnosis of scrub typhus was more common 17 (36.17%) in age group of (40-60 years) with female predominance 32 (68.08%). Most patients (70.15%) were of above 40 years. Fever 47 (100%), asthenia 40 (85.10%), generalized body-ache 41 (87.23%), anorexia 46 (97.87%) and headache 39 (82.97%) were present in most of our patients at sometime during their illness. Respiratory dysfunction was the commonest 37 (78.72%) system dysfunction followed by renal 30 (63.82%) and hepatic 20 (42.55%) impairment. Fortunately no deaths occurred.

**Conclusions:**

Scrub typhus occurred more commonly in elderly female patients. Early empirical treatment may prevent mortality. Large studies involving whole country is needed to see real scenario of disease in this setting.

## INTRODUCTION

Acute febrile illness (AFI) is one of the most common symptoms for which patients seek medical care in our scenario. One to three percent of AFI is caused by scrub typhus in our country.^1^

It is often neglected and undiagnosed as a cause of AFI because of absence of specific clinical features. This disease is re-emerging in south East Asia and case fatality rate may reach upto 30%.^2,3,4,5^ One large study in Nepal shows that there were 831 cases of scrub typhus with 14 deaths till 2017.^6^ There seems variation in mortality from scrub typhus in Nepal in different studies ranging from 1.7% to 7.92%.7,8 In a systematic review report even after treatment median case fatality rate was 1.4 % (0-33%).^9^ There are no studies on clinical profile and outcome of adult patients with scrub typhus in Province no 1 Nepal.

Therefore, this study aimed to find out the clinical profile and outcome of hospitalized patients with scrub typhus.

## METHODS

This was a descriptive cross-sectional study carried out in the Department of Internal Medicine of Nobel Medical College, Biratnagar which is a tertiary health care center in Province 1, from 1st January 2018 to 30th December 2019 (Duration 1 year). This study was approved by the Institutional Review Committee of our medical college. All the patients of scrub typhus were enrolled in the study. Early appropriate treatment of acute infection results in good patient outcome. We use syndromic approach^10^ “hard hit initially” for treating the patients whom we suspect severe infection and those who need hospitalization. They were treated with cephalosporin and doxycycline from beginning. In this study panel of serological tests for scrub typhus, typhoid, leptospirosis, dengue fever and malaria as Acute Febrile Illness (AFI) Profile. This set of profile was affordable to most of our patients. This panel test was sent for those patients who were diagnosed as Acute Undifferentiated Febrile Illness (AUFI).We included all consecutive patients, who had diagnosis of scrub typhus serologically. Coinfection also occurs in a patient. Coinfected patients with dengue, malaria, leptospirosis, and typhoid fever were not included in the study. For the diagnosis of Scrub Typhus, we used Rapid Scrub Typhus IgG/IgM Combo Test Card which utiilizes the principle of immuno-chromatography. Detail history, physical examination, laboratory investigations and imaging studies were reviewed and recorded in a preformed structured proforma. Investigations included basic test parameters like complete blood counts, blood sugar, renal profile, liver function tests, chest radiograph and ultrasonography of abdomen and pelvis. Other investigations were done as per need and affordability. Operational definitions for organ system dysfunction were based upon simple available clinicolaboratory profiles and imaging. Respiratory, cardiovascular and central nervous system dysfunction were defined as any cardinal symptom that was significantly troublesome and clinically significant during illness.

Collected data were entered in Microsoft Excel 2007 and converted it into Statistical Package for Social Science (SPSS) 11.5 Version for statistical analysis. For descriptive statistics, percentage was calculated along with graphical and tabular presentation.

## RESULTS

During the one year period there were altogether 47 cases of serologically diagnosed scrub typhus patients. The results are depicted below.

Regarding age group of the patients very few of them at the extremes of their age were affected. More commonly affected age group was 40-60. Scrub typhus can affect any sex but Females were most commonly affected 32 (68.08%) by this disease. Most of them 38 (80.85%) were agricultural workers (Table 1). When people become sick, first they approach local level health facility for treatment which may reduce their symptoms. Therefore patients come late to the tertiary hospital. Most of the patients presented to our hospital after a week of appearance of their symptoms. Our hospital is located at the capital of province no 1 in Biratnagar Morang district. Most of the patients were affected during July to September, largest number in August (Table 2).

**Table 1:** Socio-demographic characteristic of the patients.

Characteristics	Categories	n(%)
Age	0-20	2 (4.2)
	20-40	12 (25.53)
	40-60	17 (36.17)
	60-80	14 (29.78)
	>80	2 (4.2)
Sex	Male	15 (31.91)
	Female	32 (68.08)
Duration of illness	Less than 7 days	10 (21.27)
	More than 7 days	37 (78.72)
Primary Occupation	Agriculture	38 (80.85)
	Professionals	6 (12.76)
	Others	3 (6.38)

**Table 2:** Monthly distribution of patients of scrub tyhpus in adults.

Months	n (%)
May	1 (2.1)
Jun	6 (12.76)
July	9 (19.14)
August	16 (34.04)
September	7 (14.89)
October	4 (8.51)
November	3 (6.38)
December	1 (2.12)

When we searched the features of infection in our patients, all the symptoms of the infections were not present at the same time. They occurred either sequentially or some disappeared or other recurred or newly appeared at any time of their illness. Fever (100%), asthenia, generalized bodyache, anorexia and headache were present in most of our patients at sometime during their illness (Table 3). Eschar present in any part of the body greatly helps us in clinical evaluation of the patients and helps us to empirically diagnose scrub typhus. Anterior chest and abdomen were common sites in our study. Careful patient examination is important because eschar may be present in groin, thigh (Table 4). In our patients the comorbidities were not significant despite being older adults. There are many reports that scrub typhus affects all the systems of the body and may be complicated by severe system dysfunction even leading to death. Our study showed that respiratory dysfunction was the commonest system dysfunction which we recognized in the form of tachypnea, excessive cough, chest pain even dyspnea. Renal and hepatic impairment were common findings in scrub typhus patients. Most of them were not clinically symptomatic needing intervention and recovered during admission. Cardiovascular and central nervous system dysfunctions were minor in the form tachycardia hypotension and altered mental status. During treatment they recovered very quickly and without extra intervention.

**Table 3:** Chief complaint of the patients.

Characteristics	n (%)
Fever	47 (100)
Extreme weakness	40 (85.10)
Fever with chills and rigors	34 (72.34)
Headache	39 (82.97)
Generalized Bodyache	41 (87.23)
Anorexia	46 (97.87)
Vomiting	20 (42.55)
Pain Abdomen	7 (14.89)
Facial swelling	38 (80.85)
Chest pain	30 (63.82)
Whole body swelling	22 (47)
Dyspnea	14 (29.27)
Cough	35 (74.4)
Eschar	14 (29.27)
Altered mental status	5 (10.63)
Rash	2 (4.25)

**Table 4:** Location of the eschar.

Location	n (%)
Groin	2 (14.2)
Anterior chest	7 (50.0)
Abdomen	4 (28.4)
Thigh	1 (7.14)

**Table 5:** System and organ dysfunction in scrub typhus patients.

System and Organ Dysfunction	n (%)
Respiratory system	37 (78.72)
Renal impairment	30 (63.82)
Hepatic impairment	20 (42.55)
Haematological impairment	10 (21.27)
Cardiovascular system	7 (14.89)
Central Nervous system	5 (10.63)

## DISCUSSION

Many studies are carried out in different settings to see clinical profile, outcome and their predictors in scrub typhus patients.^11,12,13^ This study analysed 47 serologically proven scrub typhus patients. Most of the patients were female agricultural workers who were involved in caring household animals. They were involved in field and jungle activities with bare foot and incompletely covering the body with clothes. This might have facilitated scrub typhus infection. Symptomatic profile of scrub typhus patients like fever and other cardinal features of infection in our study were similar with other studies.^14^ Most (78.73%) patients presented late after one week of initial symptom probably because in our community there are easily available local health facilities that treat fever empirically. The fever pattern and other symptoms of infection are non-specific for scrub typhus. In any infection the symptoms of the infection appear sequentially one after another or concurrently over days or weeks. Even fever appears once initially or after some days then disappears. This study considers fever as cardinal and consistent symptom of any infection. There are other symptoms of infection affecting different systems over the course of time. Local health worker and even clinician were confused with disappearance of the fever while at the same time other symptoms of infection still persist in patients. This situation leads us to think that patients are cured of the disease. That is why patients present late in tertiary care center. Same thing existed in scrub typhus patients also. But in this hospital, we use syndromic approach to treat patients with cephalosporin and doxycycline before doing fever profile testing in laboratory. This is simple and cheap treatment protocol not missing common infectious disease including scrub typhus. Probably this might have prevented severe complications and death in our setting. During this study, we found that many patients have systemic symptoms of scrub typhus. But elderly patients with renal impairment have more serious disease requiring extra care and attention. This is mentioned in other studies as well.^15,16^ They had more systemic symptoms than other patients who did not have renal impairment. But no any patients required haemodialysis. Respiratory symptoms recovered with treatment. Patients with history of COPD and or renal impairment had more cough and dyspnea. Fortunately patients with hepatic impairment did not need extra hepatic care and recovered with treatment. The findings corroborated with other studies.^17,18^ Hematological impairment like thrombocytopenia did not require platelets replacement. No patients had clinical evidence of bleeding and death. Similar results were obtained by other researchers.^13,17^ Many literatures have mentioned severe haemodynamic complications in scrub typhus patients specially in children but surprisingly our patients required no vasopressor support and fluid replacement therapy. Acute delirium in the form of altered mentation found few elderly patients which did not require investigation and treatment. Here what we assume “Hit Hard Initially” probably treated the cause of the scrub typhus that lead to regression of disease severity and symptoms. Treatment of some symptoms was frustrating like anorexia, asthenia, headache and mild body swelling that really created patient dissatisfaction. After this study we think that the scrub typhus needs to consider in patients with AUFI. Early empirical treatment with cephalosporin and doxycycline may prevent complication and death. This study was small and confined to a tertiary care center in province 1. We could not find the predictors of mortality in scrub typhus patients. We recommend using syndromic approach in treating patients with acute undifferentiated febrile illness. We also recommend carrying out large scale prospective study that includes whole nation.

## CONCLUSIONS

In this small study initial empiric treatment “Syndromic Approach” in treating patients resulted in good clinical outcome. No death occurred. Early empirical treatment may prevent mortality. The study on large number of patients from the different parts of the country may be required to know more about the status of the disease in our setting.

## Conflict of Interest

**None.**
